# Immunometabolic Responses after Short and Moderate Rest Intervals to Strength Exercise with and without Similar Total Volume

**DOI:** 10.3389/fphys.2016.00444

**Published:** 2016-10-25

**Authors:** Ricardo R. Agostinete, Fabrício E. Rossi, Alan José B. Magalhaes, Ana Paula R. Rocha, Sérgio S. Parmezzani, Jose Gerosa-Neto, Jason M. Cholewa, Fabio S. Lira

**Affiliations:** ^1^Department of Physiotherapy, São Paulo State University, Presidente PrudenteSão Paulo, Brazil; ^2^Department of Physical Education, Institute of Bioscience, São Paulo State UniversityRio Claro, Brazil; ^3^Exercise and Immunometabolism Research Group, Department of Physical Education, São Paulo State UniversityPresidente Prudente, Brazil; ^4^Department of Kinesiology, Recreation, and Sport Studies, Coastal Carolina UniversityConway, SC, USA

**Keywords:** strength exercise, intervals of recovery, testosterone, inflammation, metabolism

## Abstract

This study investigated the influence of short and moderate intervals of recovery with and without equated volume during an acute bout exhaustive strength exercise on metabolic, hormonal and inflammatory responses in healthy adults. Eight physically active men (23.5 ± 3.1) performed three randomized sequences: Short (70% of 1 RM with 30 s of rest); Moderate (70% of 1 RM with 90 s of rest); and Volume-Equated Short (70% of 1 RM with 30 s of rest between sets with a repetition volume equal to that performed in Moderate). All sequences of exercises were performed until movement failure in the squat, bench press and T-bar row exercises, respectively. Glucose, lactate, testosterone, IL-6, IL-10, IL-1ra, and MCP-1 levels were assessed at rest, immediate post-exercise, and 1 h post. There was a main effect of time for testosterone (*p* < 0.001). The *post hoc* indicated differences between post-exercise and rest and post-1 h and post-exercise (*p* < 0.001). Lactate increased post-exercise when compared to pre and post-1 h (*p* < 0.001) and maintained higher post-1 h in relation to rest. IL-6 was greater post-exercise than rest (*p* = 0.045) and post-1 h and rest (*p* = 0.020). IL-10 was greater post-exercise (*p* = 0.007) and post-1 h (*p* = 0.002) than rest. IL-1ra increased post-exercise in relation to rest (*p* = 0.003) and MCP-1 was greater post-exercise than rest (*p* < 0.001) and post-1 h (*p* = 0.043). There were no significant differences between conditions or interaction. Thus, both short and moderate intervals of recovery induced greater metabolic, hormonal and inflammatory responses after acute bout of exhaustive strength exercise in healthy adult.

## Introduction

It is well established in the literature that consistent resistance training can increase muscle strength via neural adaptations (Melibeu Bentes et al., 2012) and morphological adaptations in muscle size induced by elevated systemic hormones (Kraemer and Ratamess, 2005), mechanical loading, and metabolic stress (Schoenfeld, 2010). Coaches and practitioners will manipulate variables such as volume, intensity, repetition speed, and inter-set rest to optimize the specific adaptation. The manipulation of these training variables will also alter the immunometabolic response to training, thereby affecting the desired adaptation.

Metabolic stressors associated with resistance exercise result in an acute increase in systemic anabolic hormone concentrations and an inflammatory response (Ball, 2015). Acute bouts of moderate intensity exercise (70% of 1 repetition maximum—RM) have been shown to elevate post-exercise testosterone concentrations (Rietjens et al., 2015), stimulating protein synthesis, inhibiting protein degradation, and potentially promoting muscle hypertrophy (Vingren et al., 2010). Acute exercise also stimulates interleukin-(IL) 6, which when secreted from muscle tissue promotes an anti-inflammatory response by inhibiting tumor necrosis factor alpha (TNF-α), IL-1, IL-8, and IL-12 (Pedersen and Febbraio, 2005), and increasing release of IL-10, IL1-ra (Petersen and Pedersen, 2005). IL-6 initiates the recovery process by modulating muscle regulatory genes (i.e., MyoD; Warren et al., 2002; Gleeson and Bishop, 2005; Tidball, 2005) and activating muscle satellite cells (Serrano et al., 2008), and therefore may play a role in the hypertrophic process. Additionally, IL-6 activates glucagon-like peptide-1 (GLP-1; Pal et al., 2014) and to increases AMPK and/or PI3 kinase activity which thereby increasing glucose and fatty acid uptake and oxidation (Pedersen, 2011).

Although the literature is rich with studies that have investigated the effects of varying resistance training variables on the acute hormonal response (de Salles et al., 2010; Rahimi et al., 2010; Jambassi Filho et al., 2013) very few studies have measured the immunological response to training variable manipulation. Uchida et al. (2009) reported no differences in the immunological response to varying intensities of bench press with 2 min of rest and matched volume loads (sets × reps × load). Phillips et al. (2010) reported greater post-exercise IL-6 concentrations with 65% 1 RM compared to 85% 1 RM and 2 min recovery intervals. Our group recently (Rossi et al., 2016) investigated the differences between short (30 s) and moderate (90 s) intervals of recovery during an acute bout of exhaustive strength exercise with 4 sets of 70% of 1 RM in squat and bench press. The volume load performed was significantly greater (~27%) in the moderate rest condition and IL-6 increased only in the moderate rest condition.

The results of Rossi et al. (2016) and Phillips et al. (2010) suggest that longer recovery intervals and higher total volume load contributes to resistance exercise induced IL-6, however, the effect of different recovery intervals with similar volumes on the acute inflammatory response is unknown. Therefore, the aim of this study was to investigate the influence of short (30 s) and moderate (90 s) intervals of recovery during an acute bout of exhaustive strength exercise on performance, metabolic, hormonal, and inflammatory responses with and without matched total volume loads in healthy adults. We hypothesized that the higher metabolic demand during matched volume condition with short recovery intervals would lead to greater metabolic, hormonal and inflammatory response.

## Methods

### Experimental approach to the problem

**Figure 1:**
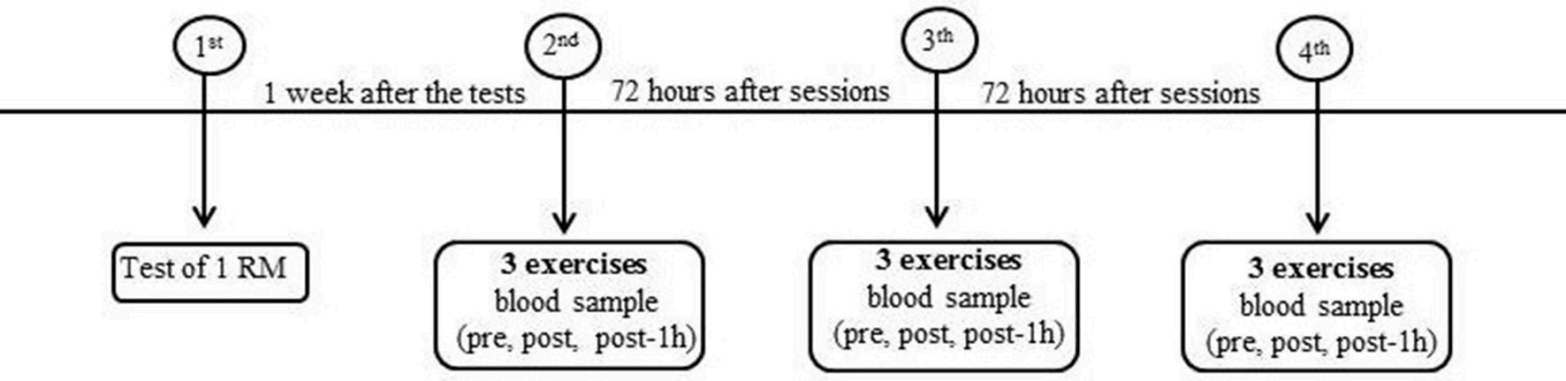
**Study design**.

To investigate the effects of two different inter-set recovery intervals (30 and 90 s) on the metabolic, hormonal and inflammatory response, data was collected using a randomized and counterbalanced within-subjects design. One repetition maximum (1 RM) testing was conducted on nonconsecutive days 1 week before the exercise protocols for all subjects. During the exercise sessions blood samples were collected pre-, post- and 1 h post-exercise (Figure 1). The experimental protocol was carried out at the resistance training facility at the Univ. Estadual Paulista (UNESP) in Presidente Prudente-SP, Brazil.

### Subjects

Eight physically active males voluntarily participated in this study. Inclusion criteria for participation in the study were: not participating in regimented strength training during the previous 6 months (1); age between 18 and 30 years; no contraindications involving the cardiovascular system, muscles, joints, or bones of the lower limbs that may limit the practice of strength training; and, signing the consent form. This study was conducted in accordance with the Helsinki Declaration. This study was approved by the Ethics Committee (number: CAAE 22793414.7.0000.5402).

### Procedures

#### Anthropometric measurements, body composition and dietary intake assessment

Anthropometry consisted of body weight and height measurements. Body weight was measured using an electronic scale (Filizola PL 50, Filizola Ltda., Brazil), with a precision of 0.1 kg. Height was measured on a fixed stadiometer of the Sanny brand, with an accuracy of 0.1 cm and a length of 2.20 m. Body composition was estimated using a DXA scanner, version 4.7 (General Electric Healthcare, Lunar DPX-NT; England). The subjects were positioned in a supine position and stayed immobile throughout the examination. Fat mass and lean mass were evaluated and expressed in percentage values. All measurements were carried out at the University laboratory in a temperature-controlled room. Each morning before the beginning of the measurements the equipment was calibrated by the same researcher according to the manufacturer's instructions.

Diet was not standardized between subjects, however subjects were instructed to consume the same breakfast each 3 days of testing. The volunteers were instructed how to complete the food records and were required to record all foods consumed on the morning of each testing session and 2 days before each test. The average between the 3 days was calculated for energy intake (express in Kcal) and macronutrient distribution (in grams). Nutrition data was analyzed using the NutWin software, version 1.5 (Programa de Apoio à Nutrição, Universidade Federal de São Paulo, Brazil, 2002).

### Test of one maximum repetition (1 RM)

One repetition maximum testing in the order of squat, bench press and T-bar row occurred 1 week prior to the experimental protocols on Monday and Thursday, and the best attempt from the two sessions was recorded as the 1 RM. All participants performed a warm-up (jogging) for 5 min and then one set of ten repetitions of the respective exercise at approximately 50% of the 1 RM prior to 1 RM testing. The load was increased gradually (10–15%) during the test until the participants were no longer able to complete the movement, and 3–5 attempts were allowed (Thompson et al., 2013). An interval of 3 to 5 min of rest was provided between attempts (Thompson et al., 2013). No rest was allowed between the concentric and eccentric phases of the movement, and the participants were encouraged verbally to exert a maximum effort. Two fitness professionals oversaw all testing sessions.

### Experimental protocol

**Figure 2:**
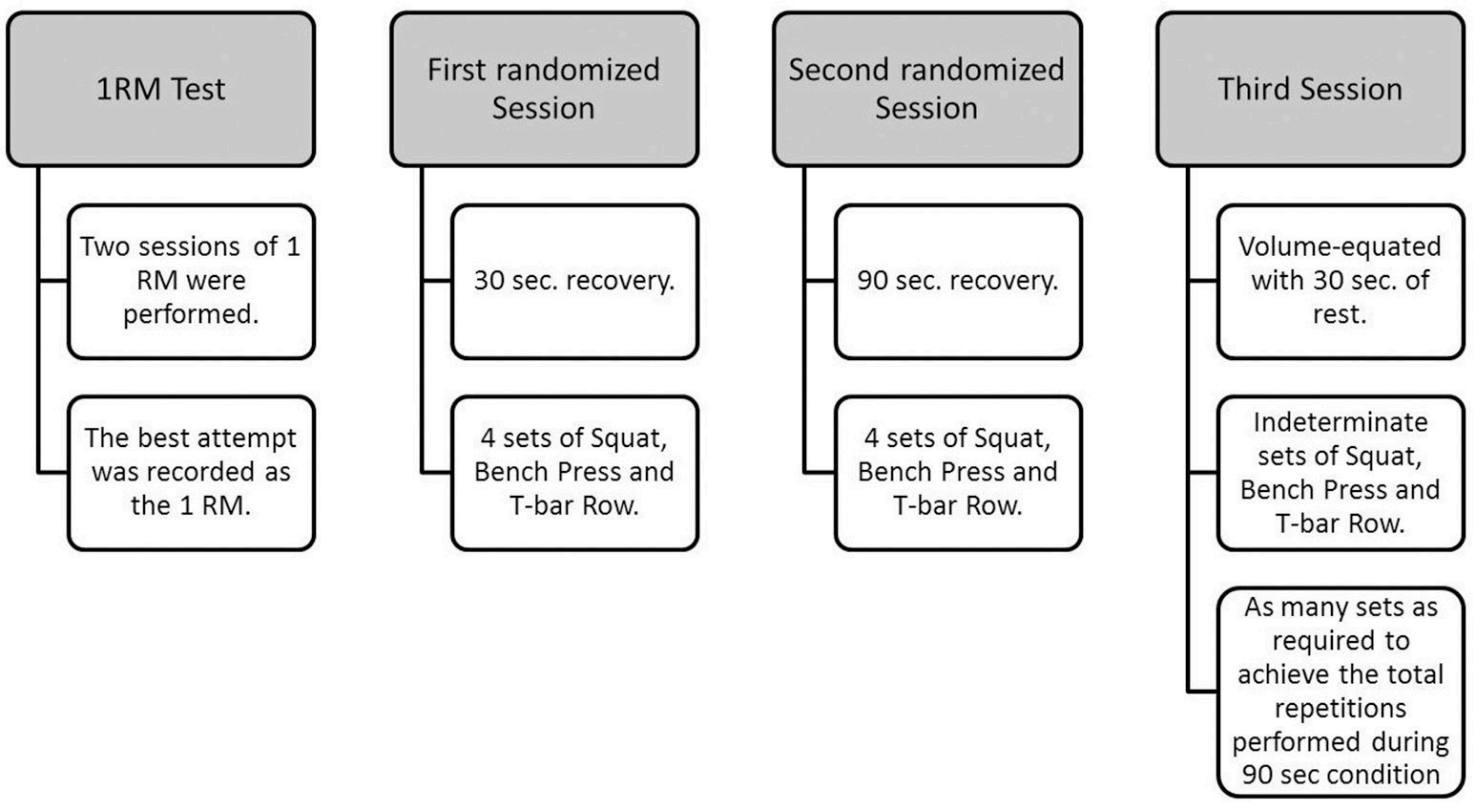
**Experimental protocol**.

One week after the 1 RM test subjects performed two randomized exercise sessions of either short (30 s recovery between sets) or moderate (90 s recovery between sets) recovery intervals followed by a third volume-equated exercise session. For the first two conditions the subjects performed 4 sets of squat, bench press and T-bar row with 70% 1 RM, respectively. On the third day the volume-equated session was carried out with 30 s of rest and 70% 1 RM. To equate the volume subjects were required to perform extra sets until the total number of repetitions for each exercise was equal to that accomplished during the moderate condition. All the sequences of exercises were performed until movement failure for each exercise with normal speed (1-s eccentric and 1-s concentric actions with 1-s rest between each repetition). The total number of repetitions performed was recorded for each set of each exercise and for all sequences, and used to analyze workload and performance. During the exercise sessions, subjects were verbally encouraged to perform all sets to exhaustion in each exercise and the length of time between experimental tests days were separated by at least 72 h (Figure 2).

### Blood samples and analysis

Blood samples were collected at rest, post-exercise and 1-h post-exercise. The blood samples (10 mL) were immediately allocated into three 3 mL vacutainer tubes. BD Vacutainer® Fluoreto/EDTA tubes were used to measure glucose and lactate; Gel BD SST® II Advance® tubes were used for serum separation; and BD Vacutainer® EDTA K2 (Becton Dickinson, BD, Juiz de Fora, MG, Brazil) tubes were used for plasma separation. The tubes were centrifuged at 3000 RPM for 15 min at 4°C, and samples were stored at −20°C until analysis. Glucose and lactate were assessed through commercial enzymatic kit (Labtest®, São Paulo, Brazil). Testostorone was assessed by a colorimetric method with a commercial kit (Monobind inc®, 100 N. Pointe Dr., Lake Forest, CA 92630 USA). Cytokines IL-6 and IL-10 were assessed using ELISA commercial kits (affimetrix/eBioscience, Ambriex S/A, Rua Traipu, 125, São Paulo—SP 01235-000, Brasil), IL-1ra and MCP-1 were assessed using ELISA commercial kits (R&D Systems, Inc. 614 McKinley Place NE Minneapolis, MN 55413). To eliminate inter-assay variance, all samples were analyzed in identical runs resulting in an intra-assay variance of <7%. Standard curve range for IL-1ra (9.8–2500 pg/mL), IL-6 (1.6–200 pg/mL) and IL-10 (2.3–300 pg/mL), MCP-1 (15.6–1000 pg/mL), glucose reference standard was 100 mg/dL, and for lactate reference standard was 40 mg/dL.

### Statistical analysis

The comparison of the maximum number of repetitions in each series were analyzed with a one-way repeated measurements ANOVA and the differences in the inflammatory, metabolic, and hormonal responses were analyzed by performing a two-way repeated measures of ANOVA (condition × time). When a significant difference in group or interaction was observed, a Tukey *post hoc* test was conducted. For all measured variables the estimated sphericity was verified according to Mauchly's *W*-test and the Greenhouse–Geisser correction was used when necessary. Effect sizes were calculated and defined as small (0.20), medium (0.50), and large (0.80). The test-retest reliability coefficient for 1 RM was calculated and *r*-values were presented. Statistical significance was set at *p* < 0.05. The data were analyzed using the Biostat (version 5.0).

## Results

Table 1 presents the mean values of age, height, weight, fat mass, and lean mass in percentage and dietary intake of the sample. There were no significant differences in total food intake (expressed in kcal) between conditions (*p* = 0.216) or macronutrient distribution (Carbohydrates: *p* = 0.112; Protein: *p* = 0.750; Lipids: *p* = 0.944).

**Table 1:** **General characteristics of the sample, strength test, dietary intake and macronutrient distribution**.

**Variables**	**Mean ± *SD* (*n* = 8)**			
Age (years)	23.5 ± 3.1			
Height (cm)	180.8 ± 7.6			
Weight (Kg)	80.7 ± 6.4			
Fat mass (%)	22.5 ± 3.7			
Fat free mass (%)	58.4 ± 4.8			
**Strength test**	**Test**	**Re-test**	**1 RM**	***r*****-value**
Squat (Kg)	170 ± 56	178 ± 50	178 ± 50	0.90
Bench press (Kg)	60 ± 11	64 ± 11	64 ± 11	0.88
T-bar row (Kg)	64 ± 16	65 ± 16	65 ± 14	0.88
**Dietary intake**	**30 s**	**90 s**	**Volume-equated**	
Total food intake (Kcal)	1592 ± 243.0	1754 ± 298.0	1496 ± 313.0	
Carbohydrates (g)	205.1 ± 46.4	234.9 ± 56.6	179.6 ± 46.7	
Protein (g)	77.7 ± 17.5	77.4 ± 24.3	85.4 ± 28.1	
Lipids (g)	44.9 ± 15.1	44.3 ± 9.0	42.7 ± 14.7	

Table 2 showed the comparison between 30, 90 s and volume-equated on the total repetitions and total volume performed. There were statistically significant differences between 90-s, volume-equated and 30 s for maximal number of repetitions (*p* = 0.002). There was a statistically significant decrement in repetitions performed between the first and second set for all conditions (*p* < 0.05).

**Table 2:** **Comparison between 30, 90 s and volume-equated on the total repetitions and total volume performed**.

	**Set 1**	**Set 2**	**Set 3**	**Set 4**	**Set 5**	**Set 6**	**Set 7**	**Set 8**	**Total**
**TOTAL REPETITIONS**									
30 s	46.8 ± 4.1	17.1 ± 2.9	10.5 ± 3.1	7.4 ± 2.2	NA	NA	NA	NA	81.8 ± 7.5
90 s	51.4 ± 8.6	26.9 ± 5.6^a^	17.1 ± 3.6	13.5 ± 3.4^a^	NA	NA	NA	NA	109.0 ± 18.3^a^
Volume-equated	59 ± 6.3	22.9 ± 5.9	11.0 ± 3.1	7.0 ± 3.5^b^	4.0 ± 2.6	3.0 ± 1.7	2.0 ± 1.8	0 ± 0.7	109.0 ± 18.3^a^
**TOTAL VOLUME**									
30 s	3931 ± 1355.8	1400.4 ± 326.6	887.7 ± 325.4	639.4 ± 216.7	NA	NA	NA	NA	6858 ± 1757.6
90 s	4184 ± 1226.5	2166.2 ± 780.5^a^	1429.1 ± 513.5^a^	1164.4 ± 400.3^a^	NA	NA	NA	NA	8944 ± 2711.2
Volume-equated	4970 ± 1503.9	1754 ± 463.8	762 ± 370.3^b^	572 ± 442.8^b^	415 ± 241.8	246 ± 237	211 ± 203	240 ± 279.3	8944 ± 2712.5

Total repetitions and total volume performed (repetition^*^kg) in four sets at 70% 1 RM in the half-squat exercise plus bench press plus T-bar Row.

a *Statistically significant difference from 30 s*.

b *Statistically significant difference from 90 s*.

**Figure 3:**
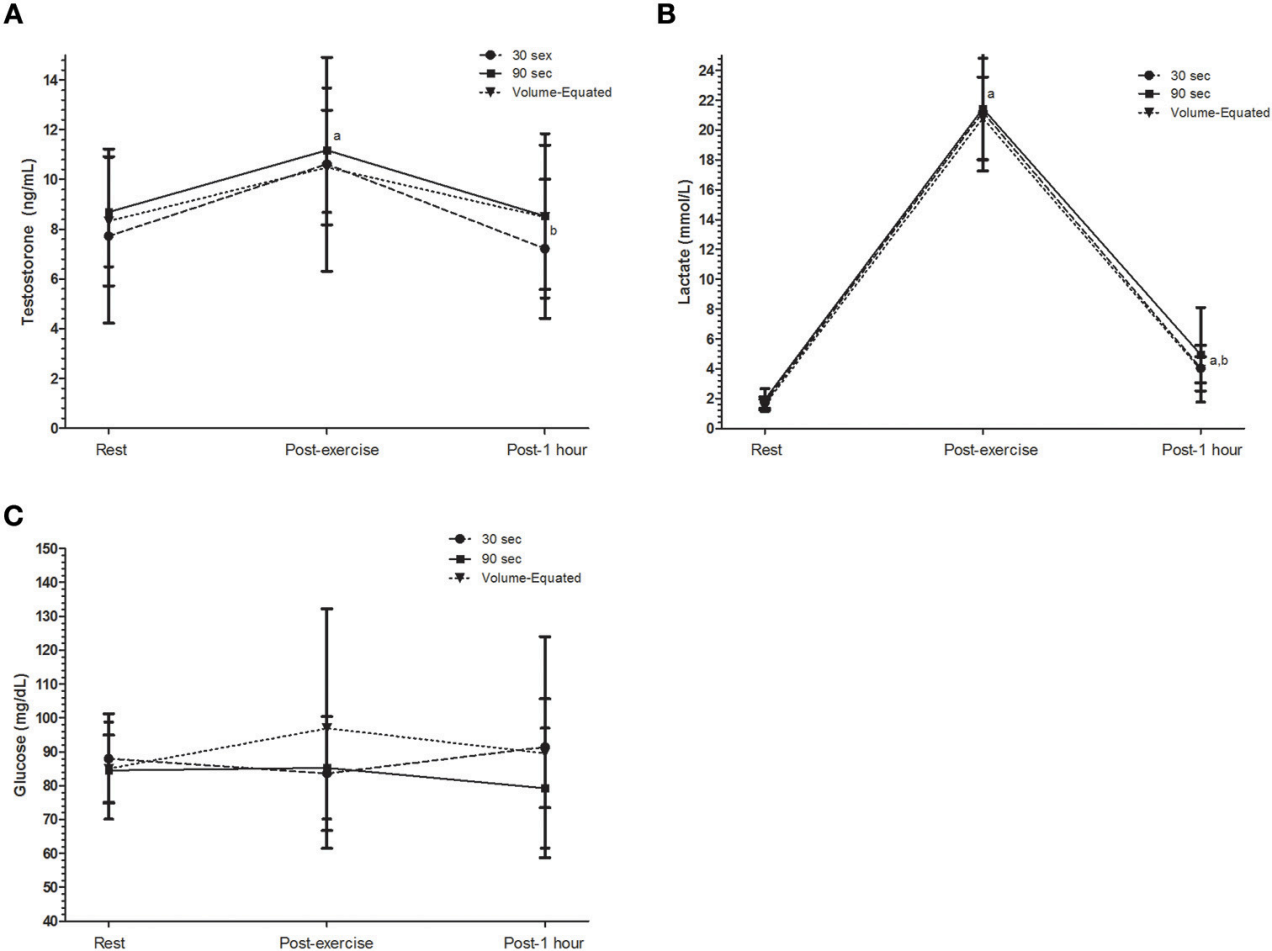
**Metabolic and hormonal parameters. (A)** Testosterone (mg/ml); **(B)** Lactate (mmol/L); **(C)** Glucose (mg/dL); 30 s (30 s of interval between sets); 90 s (90 s of interval between sets).

Figure 3 presents the metabolic and hormonal variables for the three experimental conditions.

For testosterone there was a main effect for time (*p* < 0.001). The *post hoc* indicated differences between post-exercise and rest (*p* < 0.001), and post-1 h and post-exercise (*p* < 0.001). Effect sizes ranged from moderate to large in the 30 s (0.74), and large in the 90 s and volume-equated, respectively (0.9 to 1.06). For lactate there was a main effect for time. Post-exercise was greater than pre and post-1 h (*p* < 0.001) and post-1 h was greater than rest. Effect sizes were largest for all groups (>0.80). There were no effects for time in glucose and statistically significant difference between conditions or interaction.

**Figure 4:**
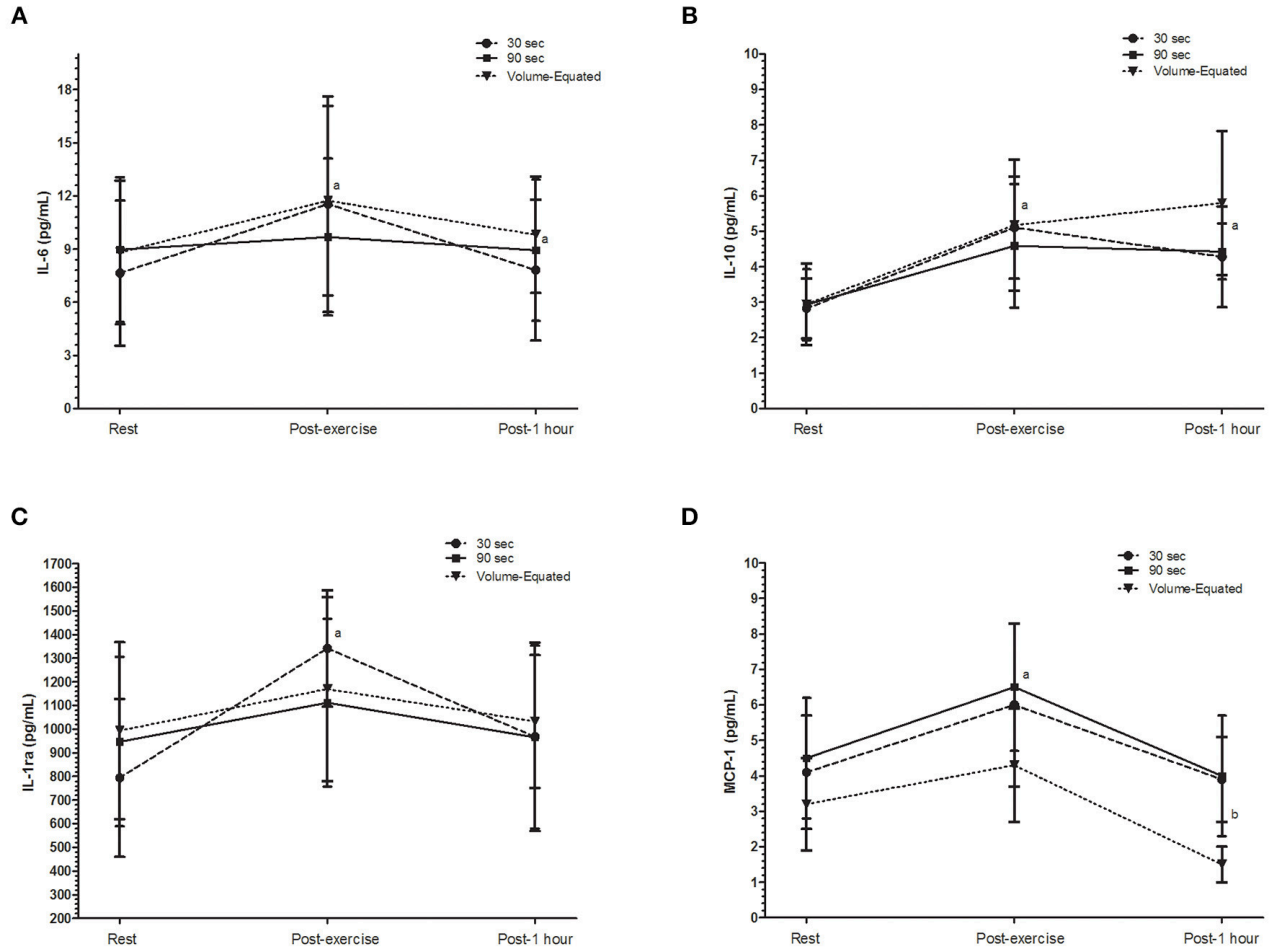
**Inflammatory parameters. (A)** Interleukin-6, IL-6 (pg/mL); **(B)** Interleukin-10, IL-10 (pg/mL); **(C)** Interleukin 1 receptor antagonist, IL-1ra (pg/mL); **(D)** Monocyte Chemoattractant Protein-1, MCP-1 (pg/mL); 30 s (30 s of interval between sets); 90 s (90 s of interval between sets).

Figure 4 presents the inflammatory responses for the three experimental conditions.

For IL-6, there was a main effect for time. The *post hoc* indicated differences between post-exercise and rest (*p* = 0.045) and post-1 h and rest (*p* = 0.020). Effect sizes ranged from moderate to large in the 30 s (0.76) and volume-equated (0.62), but small for the 90 s (0.16). For IL-10, there was a main effect for time. Post-exercise was greater than rest (*p* = 0.007) and post-1 h was maintained greater than rest (*p* = 0.002). Effect sizes were largest for all groups (>0.80).

For IL-1ra, there was a main effect of time, post-exercise was greater than rest (*p* = 0.003). Effect sizes were largest for the 30 s (1.89) and moderate for the 90 s and volume-equated (0.46). For MCP-1, there was a main effect for time too. The *post hoc* showed that post-exercise was greater than rest (*p* < 0.001) and post-1 h (*p* = 0.043). Effect sizes ranged from 0.8 to 1.1. There were no significant differences between conditions or interaction (time × condition).

## Discussion

To our knowledge, this was the first study to investigate the immunometabolic response to varying rest intervals during an acute bout of strength training to fatigue with an equal volume condition. This design allows for comparison across both workload and rest periods. The main findings of the present study were that both short (30 s) and moderate (90 s) intervals of recovery, with and without similar workloads lead to increased metabolic, hormonal, and inflammatory response after acute bout of strength training in physically active subjects.

Classically, short rest periods have been prescribed when the objective is muscular endurance, whereas more moderate (60–120 s) rest periods are associated with training for hypertrophy. Several studies have demonstrated higher acute increases in anabolic hormones (e.g., testosterone, growth hormone) following training sessions with shorter rest periods between sets (Spiering et al., 2009; Fragala et al., 2011). The hormonal response to training has been suggested by some authors to promote adaptation by leading to increased protein synthesis and ultimately increasing strength and muscle hypertrophy (Kraemer and Ratamess, 2005). In our study, all exercise conditions increased lactate and testosterone concentrations that were maintained higher than rest at 1 h post exercise. Caution should be taken, however, when prescribing training for specific objectives based solely on the hormonal response, as a recent study reported no correlations between circulating post-exercise hormone concentrations and hypertrophy (Mitchell et al., 2013), but pointed to changes in intramuscular signaling (i.e., increases in androgen receptor content) as being responsible for increased protein synthesis post resistance exercise. Indeed, Schoenfeld et al. (2016) reported greater hypertrophy with longer rest periods following an 8 weeks training program; however, acute and chronic changes in circulating anabolic hormones were not measured.

Circulating inflammatory cytokines and myokines released by skeletal muscle, especially IL-6, play a role in skeletal muscle adaptation and remodeling to strength training (Begue et al., 2013). We hypothesized that the greatest IL-6 response would occur following the equal volume condition given that the volume and duration of training, as well as the number of eccentric contractions, appears to play a significant role in the IL-6 response to exercise (Peake et al., 2015). Our results did not fully support this hypothesis, as all conditions increased IL-6 post-exercise and 1 h post-exercise in relation to rest. Phillips et al. (2010) reported greater post exercise IL-6 concentrations following moderate intensity (65% 1 RM) training with 2 min of rest compared to higher intensity (85% 1 RM) training with 2 min rest. We recently compared the effects of 30- and 90-s of rest between 4 sets of squat and bench press with 70% 1 RM to failure in resistance trained men and reported greater post exercise IL-6 concentrations following the 90-s condition (Rossi et al., 2016).

A previous study conducted by our group investigated the effects of the same recovery intervals (30 vs. 90 s) during sets of heavy strength exercise (90% of 1 RM) on inflammatory and metabolic responses in recreational weightlifters and we observed that 90-s of rest increased IL-10 (Gerosa-Neto et al., 2016). In addition, Izquierdo et al. (2009) analyzed the effects of 5 sets of 10 reps until movement failure with 2 min of rest between sets and also found that IL-10 concentrations increased compared with pre-exercise, but is necessary again to highlight that in Izquierdo and Rossi's studies the discrepancy in volume performed between conditions likely influenced the results. In contrast, while there were significant main effects for time in the present study, there were no differences between groups for IL-10, IL1-ra, and MCP-1 response. The differences in training status and/or workload during the strength exercise sessions may explain the discrepancy in results between the present and previous studies as subjects in Heavens et al. (2014), and Rossi et al. (2016) were resistance trained while the subjects in the present study were not.

The anti-inflammatory cytokines, such as IL-10 minimize an immunosuppressive status and reestablished immune response (Gleeson and Bishop, 2005). This anti-inflammatory effect occurs due to the ability of IL-10 to inhibit macrophage derived cytokines such as TNFα, IL-1β, IL-6, and IL-8 (de Waal Malefyt et al., 1991; Sabat et al., 2010), by potentiating the expression of soluble TNF-receptors (sTNF-R; de Waal Malefyt et al., 1991; Steensberg et al., 2003; Sabat et al., 2010), and stimulating the release of other anti-inflammatory mediators such as IL-1ra (Joyce et al., 1994; Sabat et al., 2010). In the present study there was an increase in IL-1ra post-exercise without any differences between conditions, suggesting that resistance training in general may pose anti-inflammatory effects. Additionally, MCP-1 increased post-exercise and maintained higher concentrations 1 h post-exercise without any differences between conditions. This increase in MCP-1 was likely a response to muscle damage as this pro-inflammatory chemokine is responsible for the attraction and infiltration of monocytes as part of the phagocytosis process of muscular repair (Tidball, 2005).

In summary, both short and moderate intervals of recovery leads to increased metabolic, hormonal and inflammatory responses after an acute bout of exhaustive strength exercise in healthy adult. Limitations of this study that should be considered when interpreting the results include a small sample size and a high variance between subject responses. Second, given that the subjects were untrained a “repeated bout” effect may have occurred following the first or second training sessions that led to less muscle damage and overall immuno-endocrine response to the volume-equated condition. Unfortunately, in order to match the volume between 90 s of rest and the volume-equated condition it was impossible to include the equal-volume condition in the random order. Further research is required to investigate the effects of a volume matched condition with short rest periods on the immuno-endocrine response in trained subjects whereby the influence of a “repeated bout” effect may be less influential.

## Clinical implications

This study demonstrates to coaches and trainers that both short and moderate recovery intervals between sets promote an increased testosterone and lactate concentration and an anti-inflammatory response. Thus, both intra-set rest periods and session volume are an important variable to be considered when planning a training protocol and these combinations can potentiate the effect of exhaustive strength exercise. In addition, we speculate that the total workout in the strength exercise session promotes an immunological response associated with hypertrophy process, especially in trained subjects.

## Author contributions

RA, FR, AM, AR, SP, and JG carried out the study design, field work, data analysis and drafted the manuscript. JC performed the statistical analysis and drafted the manuscript. JC and FL made substantial contributions to conception and design, helped to interpretation of measurements and helped to draft the manuscript. All authors read and approved the final manuscript.

## Funding

FL thanks São Paulo Research Foundation-FAPESP for their support (2013/25310-2).

### Conflict of interest statement

The authors declare that the research was conducted in the absence of any commercial or financial relationships that could be construed as a potential conflict of interest.
